# Repeated Exposure to *Lutzomyia intermedia* Sand Fly Saliva Induces Local Expression of Interferon-Inducible Genes Both at the Site of Injection in Mice and in Human Blood

**DOI:** 10.1371/journal.pntd.0002627

**Published:** 2014-01-09

**Authors:** Tiffany Weinkopff, Camila I. de Oliveira, Augusto M. de Carvalho, Yazmin Hauyon-La Torre, Aline C. Muniz, Jose Carlos Miranda, Aldina Barral, Fabienne Tacchini-Cottier

**Affiliations:** 1 Department of Biochemistry, and WHO-Immunology Research and Training Center, University of Lausanne, Epalinges, Switzerland; 2 Centro de Pesquisas Goncalo Moniz, Fundacao Oswaldo Cruz, (FIOCRUZ), Salvador, Brazil; 3 Instituto Nacional de Ciência e Tecnologia de Investigação em Imunologia (iii-INCT), Salvador, Brazil; 4 National Institute of Science and Technology in Tropical Diseases (CNPq), Salvador, Brazil; 5 Servico de Imunologia, Complexo Hospitalar Universitario Prof. Edgar Santos, Universidade Federal da Bahia, Salvador, Brazil; Institut Pasteur de Tunis, Tunisia

## Abstract

During a blood meal, *Lutzomyia intermedia* sand flies transmit *Leishmania braziliensis*, a parasite causing tegumentary leishmaniasis. In experimental leishmaniasis, pre-exposure to saliva of most blood-feeding sand flies results in parasite establishment in absence of any skin damages in mice challenged with dermotropic *Leishmania* species together with saliva. In contrast, pre-immunization with *Lu. intermedia* salivary gland sonicate (SGS) results in enhanced skin inflammatory exacerbation upon co-inoculation of *Lu. intermedia* SGS and *L. braziliensis*. These data highlight potential unique features of both *L. braziliensis* and *Lu. intermedia*. In this study, we investigated the genes modulated by *Lu. intermedia* SGS immunization to understand their potential impact on the subsequent cutaneous immune response following inoculation of both SGS and *L. braziliensis*. The cellular recruitment and global gene expression profile was analyzed in mice repeatedly inoculated or not with *Lu. intermedia*. Microarray gene analysis revealed the upregulation of a distinct set of IFN-inducible genes, an immune signature not seen to the same extent in control animals. Of note this INF-inducible gene set was not induced in SGS pre-immunized mice subsequently co-inoculated with SGS and *L. braziliensis*. These data suggest the parasite prevented the upregulation of this *Lu. intermedia* saliva-related immune signature. The presence of these IFN-inducible genes was further analyzed in peripheral blood mononuclear cells (PBMCs) sampled from uninfected human individuals living in a *L. braziliensis*-endemic region of Brazil thus regularly exposed to *Lu. intermedia* bites. PBMCs were cultured in presence or absence of *Lu. intermedia* SGS. Using qRT-PCR we established that the IFN-inducible genes induced in the skin of SGS pre-immunized mice, were also upregulated by SGS in PBMCs from human individuals regularly exposed to *Lu. intermedia* bites, but not in PBMCs of control subjects. These data demonstrate that repeated exposure to *Lu. intermedia* SGS induces the expression of potentially host-protective IFN-inducible genes.

## Author Summary

Leishmaniasis is a vector-borne parasitic disease of serious public health importance. No efficient vaccine is currently available. Parasites are transmitted to mammalian hosts during sand fly bites. During this process, both parasites and sand fly salivary products are delivered into the skin. Immunization with salivary proteins from most sand fly species can protect mice against cutaneous leishmaniasis; however, immunization with sand fly saliva of *Lutzomyia intermedia* leads to aggravation of leishmaniasis. We investigated the impact of *Lutzomyia intermedia* saliva exposure on the development of immune response to *Leishmania braziliensis*, the major causative agent of tegumentary leishmaniasis in Brazil. To this end, we analyzed in mice the gene expression pattern induced by immunization with salivary gland extracts. Among the genes highly induced were the interferon-inducible genes known to contribute to resistance against parasite infections. These genes were also induced in blood cells of human individuals that were naturally pre-exposed to bites of *Lutzomyia intermedia* sand flies. Interestingly, subsequent infection with *Leishmania braziliensis* blocked the induction of these genes in mice. These data show that the induction of potentially protective genes by insect saliva can be altered by the infecting parasite. This should be considered when including salivary components in a vaccine.

## Introduction

*Leishmania* protozoan parasites induce a broad spectrum of disease including cutaneous lesions and visceral leishmaniasis the latter being fatal if not treated. *L. braziliensis* parasites can be transmitted by *Lu. intermedia* sand flies in Central and South America where they are the leading cause of American cutaneous and mucocutaneous leishmaniasis. During a blood meal, the host is exposed to a variety of sand fly factors. Sand fly saliva contains many pharmacological agents aimed at obtaining the optimal amount of blood for nutrition, egg development and survival. In addition, the proteophosphoglycan gel which is synthesized by the parasites inside the fly midgut can exacerbate cutaneous leishmaniasis [1], [2]. Individuals living in an endemic region are bitten by both uninfected and infected sand flies, and thus are repeatedly being exposed to sand fly saliva, leading progressively to the induction of an immune response to saliva. In Brazil, *Lu. intermedia* is the predominant sand fly species harboring *L. braziliensis* [3], [4] and in Corte de Pedra, Bahia, the endemic area studied in this report, both *Lu. intermedia* and *Lu. whitmani* sand fly species exist sympatrically with fluctuations reported for these populations [5].

The role of sand fly salivary factors is also important in the establishment of infection and thus the outcome of disease. Salivary factors include mediators that circumvent the host's hemostatic responses by preventing blood clotting, vasoconstriction and platelet aggregation for optimal feeding [6], [7]. Sand fly saliva is immunogenic and the immune response to salivary antigens modulates the microenvironment at the site of the bite with an impact on the development of disease. Co-inoculation of *L. major* parasites and sand fly salivary gland sonicate (SGS) from either *Phlebotomus papatasi* or *Lutzomyia longipalpis* leads to increased lesion sizes and parasite numbers [8], [9]. In contrast, several studies demonstrated that pre-immunization with *P. papatasi* SGS, individual components of SGS, or even uninfected sand fly bites followed by infection with *L. major* resulted in protection characterized by decreased lesion sizes and parasite numbers compared to controls [8], [10], [11]. These studies suggest that the immune response associated with sand fly SGS may be detrimental to the establishment of *Leishmania* infection and salivary molecules may be included in the design of a vaccine against leishmaniasis.

In contrast, pre-exposure to *Lu. intermedia* SGS surprisingly leads to enhanced disease development after infection with *L. braziliensis*. The exacerbated disease in these mice was associated with increased parasite burdens and low IFNγ/IL-4 ratios [12]. Pre-sensitization to *Lu. intermedia* SGS induced cell recruitment, an anti-SGS antibody response and a cell-mediated immune response [12], [13]. To understand the parameters involved in the increased lesion development at the site of *L. braziliensis* inoculation in mice pre-exposed to *Lu. intermedia* SGS, we examined gene expression in the skin after repeated SGS inoculations. We wanted to understand the mechanisms by which *Lu. intermedia* SGS modulates the microenvironment and how it may enhance susceptibility to *L. braziliensis* infection. The genes that were most induced in mice were further analyzed in SGS-stimulated PBMCs of healthy individuals naturally pre-exposed to *Lu. intermedia* sand fly bites.

## Materials and Methods

### Ethical Statement

For animal studies, all animal protocols were approved by the Swiss Federal Veterinary Office and experiments were performed adhering to ethical guidelines established by this office. Recommendations in the guidelines for the care and use of laboratory animals were obtained from the Department of Security and Environment of the state of Vaud, Switzerland. The protocol has been approved by the Ethics and Veterinary Office of Regulations of the state of Vaud (SAV), Switzerland under the administrative authorization number 1266-5. For human studies, written informed consent was obtained from all enrolled subjects; all procedures were approved by the Ethical Committee of the Federal University of Bahia.

### Mice

Female BALB/c mice were purchased from Charles River (Lyon, France), housed under pathogen-free conditions in the BIL Epalinges Center and used for experiments between 6–8 weeks old.

### Sand Flies and SGS Preparation

Adult *Lu. intermedia* female sand flies were captured in Corte de Pedra, Bahia, Brazil. Entomological gathering was done on private land with permission from owners for the study to be conducted on their land and within their residences. *Lu. intermedia* sand flies were morphologically identified according to the identification key proposed by Young and Duncan [14]. Sand fly salivary glands were dissected and stored in groups of 20 pairs in 20 mL NaCl (150 mM), Hepes buffer (10 mM; pH 7.4) at −70°C. Immediately before use, salivary glands were disrupted by ultrasonication in 1.5 mL conical tubes. Tubes were centrifuged at 10,000×g for 2 min, and the resultant supernatant (SGS) was used for the studies. All SGS batches were below the limit of detection for endotoxin activity (<0.01 EU/µg) using the LAL QCL-1000 assay (Lonza, Portsmouth, NH).

### Parasites and Infections

*L. braziliensis* (MHOM/BR/01/BA788 strain) parasite which does not contain the *Leishmania* RNA virus [15] was used for experiments. The parasites were maintained *in vivo* in BALB/c mice and grown *in vitro* in M199 media (GIBCO, Paisley, UK) supplemented with 10% FCS (PAA Laboratories, Pasching, Austria), 4% HEPES (Amimed) and 2% antibiotics (penicillin, streptomycin, neomycin) (GIBCO). For infections, 1×10^6^ stationary phase promastigotes with or without SGS (equivalent of 1 pair of *Lu. intermedia* salivary glands) in 10 µL PBS were injected intradermally into the ear.

### Sand Fly Saliva Immunizations

Mice were immunized with salivary gland sonicate supernatant (SGS) as previously described [13]. BALB/c mice (at least 3–5 per group) were immunized 3 times with SGS (equivalent to 1 pair of *Lu. intermedia* salivary glands) or PBS in 10 µL in the right ear at 2-week intervals. After 2 weeks, the opposing left ear was challenged with SGS (equivalent to 1 pair of *Lu. intermedia* salivary glands) in the presence or absence of 1×10^6^ stationary phase *L. braziliensis* promastigotes. Ear lesion size was monitored weekly and measured using a caliper. To determine cellular content, ears were digested 2 weeks after challenge in the left ear using 0.2 mg/mL Liberase TL (Roche, Rotkreuz, Switzerland) for 2 h at 37°C followed by FACS analysis [16].

### Flow Cytometry

For cell surface molecules, mAb 24G2 was used to block FcRs and cells were stained using α-F4/80-biotin, α-Ly6C-FITC, α-Ly6G-APC/Cy7 (clone 1A8), α-MHCII-Alexa Fluor 700 from BioLegend (San Diego, CA) and α-CD11b-eFluor 450, α-CD11c-PE/Cy5, α-DEC205-APC, α-pan-NK CD49b-PE (clone DX5) and streptavidin-PE/Cy7 from eBioscience (San Diego, CA). All cell events were acquired on an LSRII flow cytometer (BD Biosciences, San Jose, CA) and analyzed using FlowJo (Tree Star, Ashland, OR).

### Mouse Ear Pinna Processing for mRNA Isolation and Microarray Analysis

Ears were harvested 2 weeks after challenge, homogenized using a tissue lyser (Qiagen, Hilden, Germany) and mRNA was extracted by the RNeasy Plus Mini kit (Qiagen). For microarray analysis RNA was harvested from ears 2 weeks post inoculation and for each sample condition, three independent sets of 200 ng of total RNA were isolated and used as a template for probe generation using an Ambion WT expression kit (Applied Biosystems, Foster City, CA) and the cDNA was fragmented and labeled with WT DNA terminal labeling kit (Affymetrix, Santa Clara, CA). Biotinylated sense strand fragments were hybridized to Affymetrix Mouse Gene 1.0 ST GeneChips using the Hybridization Control and Hybridization Wash and Stain kits at 45°C for 18 h. The stained array was scanned using an Affymetrix GeneChip Scanner 3000 7G to generate the CEL files. The chip data were imported with Partek Genomics Suite 6.5 (Partek, Inc., St. Louis, MO), normalized and summarized using the RMA (Robust Multiarray Average) algorithm. The relative log expression was examined to ensure that the data were properly corrected by normalization and that there were no outliers. Scatter plots were generated using Matlab 2012a (MathWorks, Natick, MA) and DataGraph 3.0 (Visual Data Tools Inc., Chapel Hill, NC). To identify expression changes between genotypes, a one-way ANOVA with contrast was performed by using the methods-of-moments.

### Mouse Ear Pinna Processing for Quantitative Real-Time PCR

Quantitative real-time PCR was carried out using random 9-mers, M-MLV reverse transcriptase RNase H- (Promega, Madison, WI) and SYBR green on a LightCycler 480 system (Roche). The primer sequences are listed in Table S1. Thermal cycle conditions started with a 5 min denaturation at 95°C and 45 cycles at 95°C for 10 sec, 60°C for 10 sec and 72°C for 10 sec. The results were normalized to the housekeeping gene hypoxanthine phosphoribosyl transferase (HPRT) using the comparative threshold cycle method (2^ΔΔCT^) for relative quantification [16].

### Processing of Human Blood Samples for Quantitative Real-Time PCR

Samples used in the present study were obtained from individuals enrolled in an epidemiological survey conducted in Corte de Pedra, Brazil, an endemic region for American cutaneous leishmaniasis, where *Lu. intermedia* sand flies transmit *L. braziliensis* [5]. Details of the area and patients are described elsewhere [17]. For the present study, individuals (n = 7) were selected based on a positive ELISA for anti-*Lu. intermedia* salivary molecules; the cutoff OD values for a positive anti-*Lu. intermedia* SGS response were established using control individuals [9]. None of control individuals had history of *Leishmania* infection and all had a negative *Leishmania* skin test. For the control group, four individuals living in a non-endemic area of Salvador, Bahia were selected based on their lack of exposure to *Lu. intermedia* SGS as determined by serology using SGS-specific ELISAs.

Following Ficoll-Hypaque gradient centrifugation, peripheral blood mononuclear cells (PBMCs) were resuspended in RPMI-1640 supplemented with 2 mM L-glutamine, penicillin (100 U/mL), streptomycin (100 µg/mL) (all from Invitrogen), and heat inactivated human serum AB Rh^+^ (Sigma Chemical Co., MO). PBMCs (3×10^6^/mL) were washed two times and resuspended in complete RPMI. Cells were plated in 24-well plates (Corning Incorporated Life Sciences, Lowell, MA) and incubated at 37°C, 5% CO_2_ in the presence or not of SGS (equivalent to 1.5 pairs of salivary glands) for 72 h. Following stimulation, cells were harvested and total RNA was extracted using Trizol (Life Technologies, Rockville, MD), according to manufacturer's instructions. RNA was eluted in water and used for cDNA synthesis (ImProm-II reverse transcription system-Promega). Real-time PCR was performed on the ABI Prism 7500 (Applied Biosystems). The primer sequences are found in Table S1. Thermal cycle conditions consisted of a two-min initial incubation at 50°C followed by a 10 min denaturation at 95°C and 50 cycles at 95°C for 15 sec and 60°C for one min each. Samples were analyzed in triplicate and the comparative method was used where gene expression cycle threshold (C_t_) values were normalized to HPRT expression as determined by the equation ΔC_t_ = C_t (target gene)_−C_t (hprt)_. Fold change was determined by 2^−ΔΔCt^, where ΔΔC_t_ = ΔC_t (SGS)_−ΔC_t (medium)_ [18].

### Statistics

Statistical analysis was performed using GraphPad Prism 5 software (San Diego, CA). For murine experiments, a two-tailed Student's unpaired *t*-test was carried out. For human experiments, a nonparametric Mann-Whitney test was applied.

## Results

**Figure 1:**
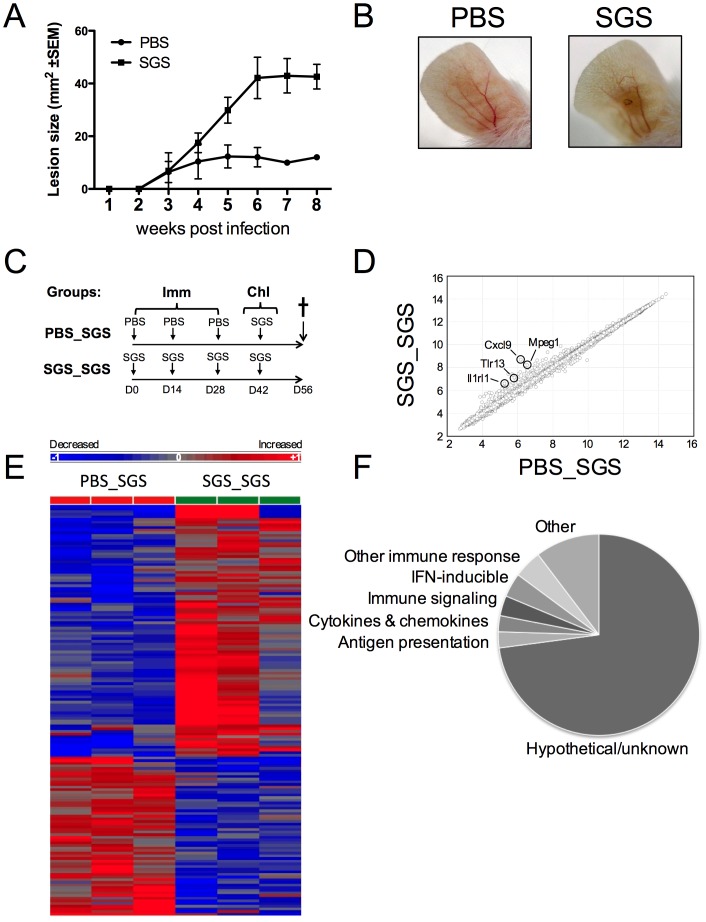
Gene categories modulated by SGS pre-immunization. BALB/c mice were inoculated 3 times in the right ear pinna every 2 wks with SGS from 1 pair of *Lu. intermedia* salivary glands and then challenged 2 wks later with *Lu. intermedia* SGS plus 1×10^6^ *L. braziliensis* parasites. (A) Lesion development was monitored weekly. Each point is the mean ±SEM of 5 animals per group. (B) Lesion images of ear pinna at 8 wks p.i. and these data are representative of two independent experiments. (C) BALB/c mice were pre-immunized (Imm) 3 times in the right ear every 2 wks with *Lu. intermedia* SGS or PBS and then challenged (Chl) in the left ear 2 wks later with *Lu. intermedia* SGS. The challenged left ears were collected after 2 wks, homogenized and gene expression was determined by microarray analysis. (D) Global significant differences in gene expression shown in log_2_ were determined comparing mice pre-immunized with SGS to those given PBS (n = 3 mice per group) and hierarchical clustering revealed genes differentially expressed >1.5× with a p value of <0.05 are presented in a heat map (E) and separated based on functional categories in a pie chart (F).

Repeated pre-exposure of BALB/c mice to *Lu. intermedia* SGS enhances susceptibility to *L. braziliensis* infection with a lesion beginning at 3 weeks post-infection (Fig. 1A and 1B) in line with previously published results [12]. Thus, we wanted to determine if differences in cellular recruitment due to pre-immunization with *Lu. intermedia* SGS prior to infection could explain the differences in disease status. Therefore, mice were repeatedly pre-immunized with SGS or inoculated with PBS and both groups were challenged with SGS in the contralateral ear. We examined the cellular infiltrate of the ear two weeks after SGS challenge, when the adaptive immune response is ongoing and the parasite has typically already established infection, despite a lack of detectable differences in lesion size. At this point, no significant differences were observed in the total number of cells, or the numbers of neutrophils, macrophages or DCs in the ears of mice pre-immunized with SGS compared to those inoculated with PBS (Fig. S1).

As a result, we hypothesized that alterations in gene expression in response to repeated exposures to *Lu. intermedia* SGS may be modulating the local skin microenvironment, impacting the innate and adaptive immune responses and thus the outcome of disease. To examine the effect of SGS pre-immunization at the inoculation site, we carried out a microarray analysis in mice that were pre-immunized with SGS or inoculated with PBS and later challenged with SGS in the opposing ear dermis. The ear pinna was processed and analyzed two weeks after the last SGS challenge (Fig. 1C).

Overall, there were few differences in the global gene expression patterns between mice that were repeatedly pre-exposed to SGS and challenged with SGS compared to those inoculated with PBS and challenged with SGS. However, hierarchical clustering analysis revealed that 95 genes were increased and 60 genes were decreased in response to SGS pre-immunization compared to control mice (Fig. 1D and 1E). Of the 155 transcripts modulated by SGS pre-immunization, only 49 transcripts have been annotated, or ascribed to a specific gene, and the rest are classified as hypothetical or unknown. Despite the majority of these genes being classified as hypothetical or unknown, many of the transcripts that were differentially expressed in response to SGS pre-sensitization are known to play a role in immune processes like antigen presentation and signaling as well as transcripts encoding for cytokines, chemokines and their receptors (Fig. 1F).

Of the 49 annotated genes differentially regulated with SGS pre-immunization, the microarray analysis revealed all but one of these annotated genes was increased upon SGS challenge in mice pre-immunized with SGS compared to those inoculated with PBS (Table 1). Of the 49 annotated genes, 4 transcripts had greater than 2-fold expression in SGS pre-exposed mice compared to controls; the gene most highly expressed in SGS pre-immunized mice compared to PBS-inoculated animals was CXCL9. Mpeg1, a transcript indicative of macrophage presence, as well as IL-1rl1 and TLR13 which are members of the toll-like superfamily of receptors, were also significantly elevated in response to SGS challenge in SGS pre-exposed mice compared to controls. The microarray results revealed an especially high frequency (14.3% of the annotated genes) of genes induced in response to SGS pre-immunization to be IFN-inducible genes including immunity-related GTPases (IRGs) and guanylate-binding proteins (GBPs) [19]–[25].

**Table 1:** Genes differentially expressed in response to SGS pre-immunization.

Gene Symbol	Description	Fold Change	p-value
**Antigen Presentation**			
CD74	CD74 antigen (invariant chain)	1.65	0.018
H2-gs10	MHCI like protein	1.72	0.003
H2Q6	Histocompatibility 2	1.61	0.009
Tap1	Transporter 1	1.51	0.0006
**Chemokine, cytokines and their receptors**			
CXCL9 (MIG)	Chemokine ligand 9	5.72	0.037
IL-1rl1 (ST2)	IL-1 receptor-like 1	2.58	<0.05
IL-7r (CD127)	IL-7 receptor	1.53	0.017
IL-10rα	IL-10 receptor α	1.69	0.016
**Immune response signaling**			
CD180	CD180 antigen	1.99	0.034
Sfpi1 (PU.1)	SFFV proviral integration 1	1.54	0.038
Sla	Src-like adaptor	1.55	0.027
Stat1	Signal transducer and activator of transcription 1	1.51	0.017
TLR13	Toll-like receptor 13	2.48	0.049
**IFN-inducible genes**			
Gbp6	Guanylate-binding protein 6	1.74	0.031
Gpb8	Guanylate-binding protein 8	1.86	0.037
Ifit1	IFN-induced protein	1.62	0.007
Iigp1	IFN-inducible GTPase	3.38	0.049
Irgm1	Immunity-related GTPase	1.65	0.033
Irgm2	Immunity-related GTPase	2.16	0.027
**Other immune response genes**			
Aif1	Allograft inflammatory factor 1	1.84	0.012
Chi3l1 (Ym1)	Chitinase 3-like 1	1.77	0.005
Igsf6	Immunoglobulin superfamily member	1.98	0.044
Klrd1	Killer cell lectin-like receptor	1.66	0.030
Mpeg1	Macrophage expressed gene	3.23	0.044
Nkg7	Natural killer cell group 7	1.67	0.040
Pdcd1lg	Programmed cell death 1 ligand 2	1.96	0.035
**Other genes**			
Apobec1	Apolipoprotein B	1.65	0.021
Atp8b4	ATPase 8B	1.70	0.004
Dpep2	Dipeptidase 2	1.52	0.040
F10	Coagulation factor X	1.88	0.038
Fyb	FYN binding protein	1.94	0.010
Havcr2	Hepatitis A virus cellular receptor	1.51	0.038
Lgals3bp	Lectin, galactoside-binding	1.50	0.042
Mir203	microRNA 203	−1.53	0.022
Mrgpra9	MAS-related GPR	1.54	0.031
Ms4a4b	Membrane-spanning 4 domains	1.99	0.020
Myo1f	Myosin 1F	1.65	0.023
Naaa	N-acylethanolamine acid amidase	1.52	0.033
Ptprc	Protein tyrosine phosphatase receptor 1	1.91	0.046
Samhd1	SAM domain and HD domain	1.51	0.048
Slfn1	Schlafen 1	1.76	0.030
Sp110	Nuclear body protein	1.57	<0.05

**Figure 2:**
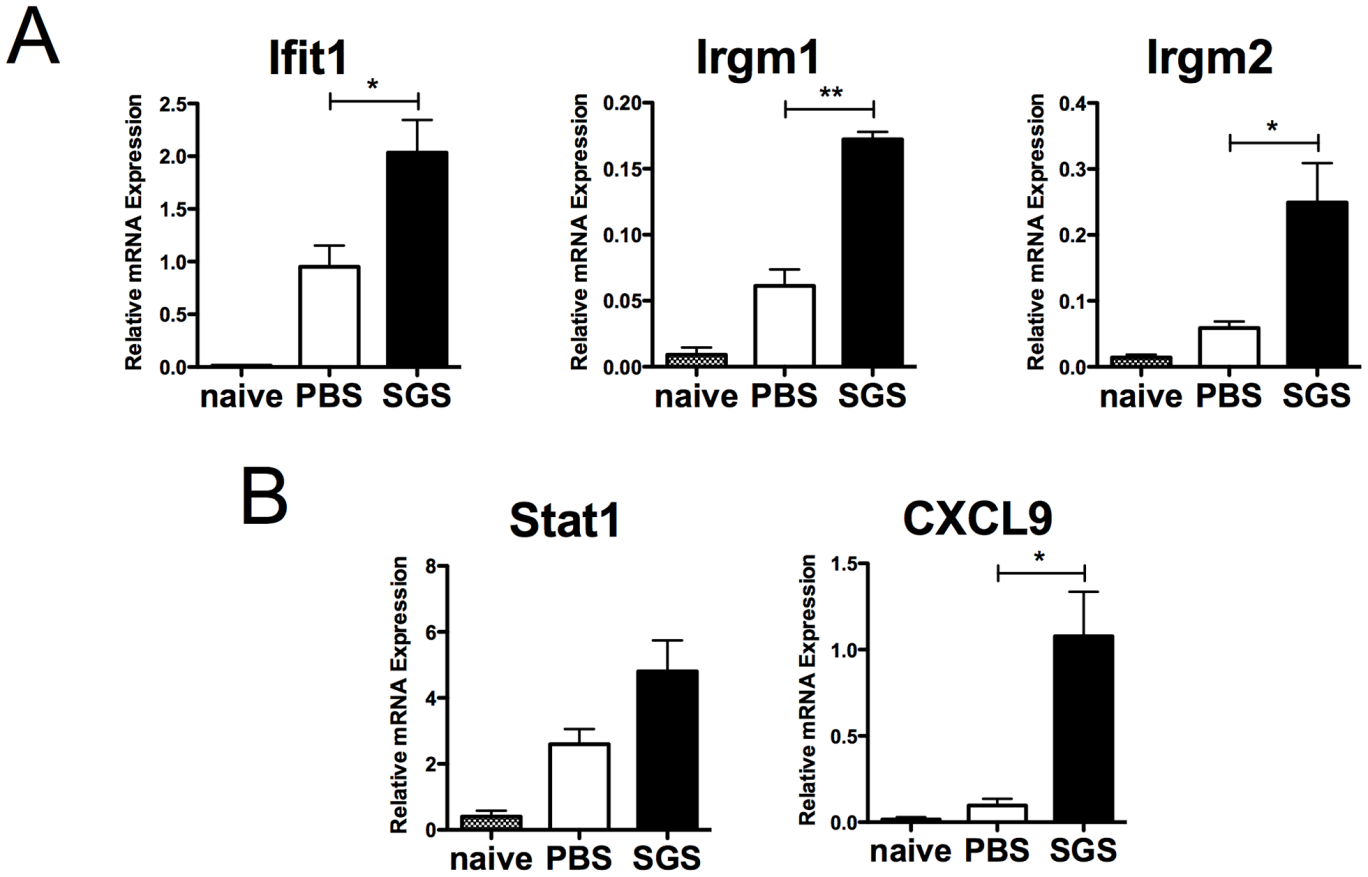
IFN-inducible genes are upregulated in response to SGS pre-immunization. BALB/c mice were inoculated 3 times in the right ear every 2 wks with *Lu. intermedia* SGS or PBS and then challenged in the left ear 2 wks later with *Lu. intermedia* SGS. The left ears were collected 2 wks after SGS challenge, homogenized and the expression of IFN-inducible genes such as (A) Ifit1, Irgm1 and Irgm2, and the expression of IFN-related genes like (B) Stat1 and CXCL9 was determined by real-time quantitative PCR normalized relative to HPRT mRNA levels. Similar analysis of ear pinna of naïve mice that did not receive any injections and were not challenged with SGS is also shown. Data are an independent biological replicate to the microarray analysis; relative mRNA expression normalized to the housekeeping gene HPRT is presented as the mean +SEM with 3–5 mice per group; ** p<0.005, * p<0.05 by Student's *t*-test.

Given the surprisingly large proportion of the modulation of IFN-inducible genes in mice pre-immunized with SGS compared to control mice, we carried out real-time qPCR for IFN-inducible genes as well as genes associated with IFN-induced responses on a biological replicate experiment to confirm the findings of the microarray analysis. Cells from mice pre-sensitized with SGS and challenged with SGS had a higher expression of Ifit1, Irgm1 and Irgm2 compared to mice inoculated with PBS and challenged with SGS. Of note, despite these differences, challenge with SGS in mice pre-immunized or not with SGS had a higher expression of these genes compared to naïve mice, suggesting SGS inoculation alone can already induce this gene family (Fig. 2A). In addition, pre-immunization with SGS also led to an increased expression of Stat1, a signaling molecule responsible for the subsequent expression of IFN-inducible genes. CXCL9, a chemokine involved in T cell migration induced by IFNγ, was also expressed at higher levels in SGS pre-exposed mice compared to control mice (Fig. 2B).

**Figure 3:**
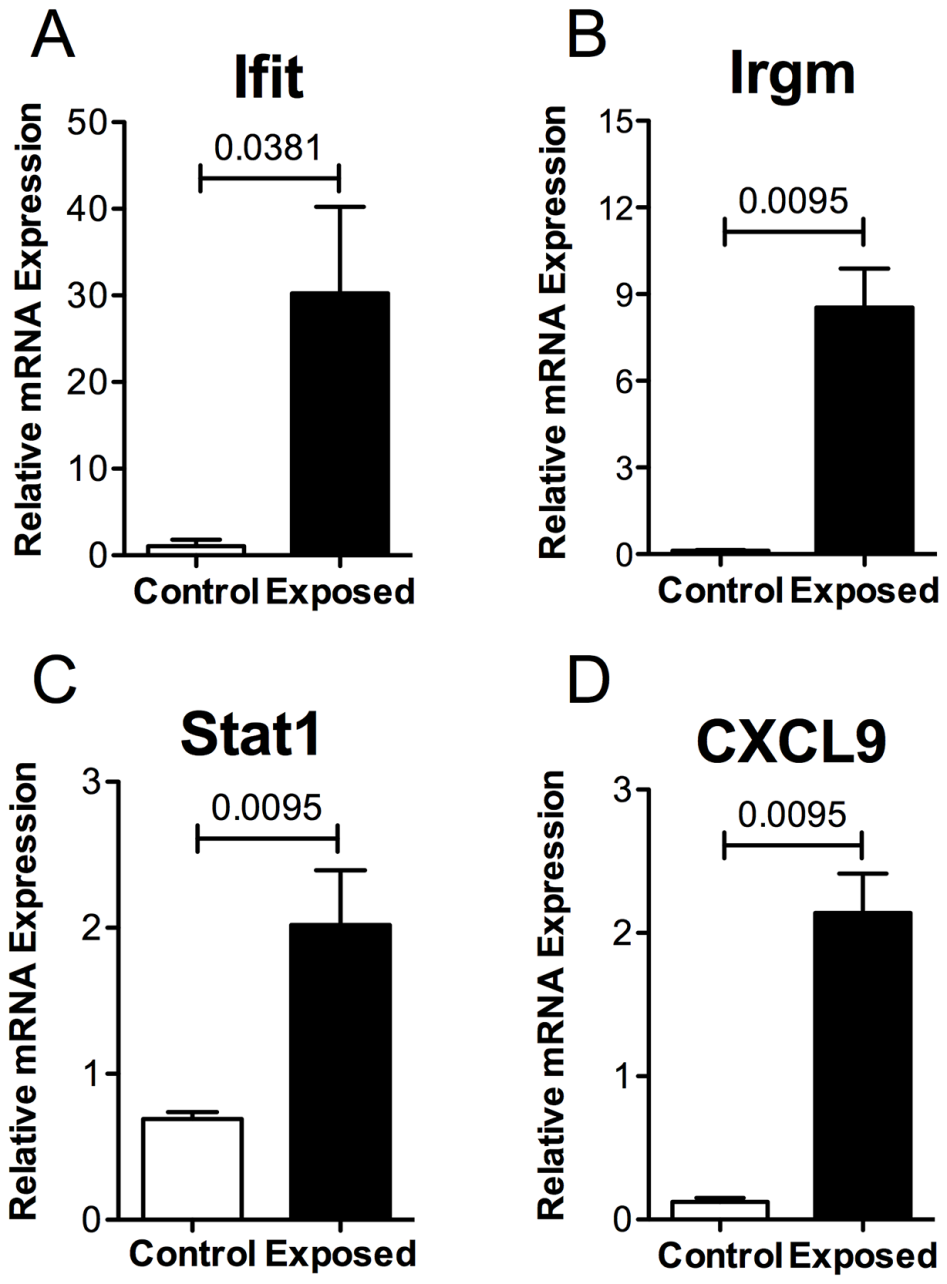
IFN-inducible genes are upregulated in human PBMCs by individuals exposed to sand fly bites. PBMCs from people exposed to *Lu. intermedia* bites living in an endemic area who expressed high anti-SGS antibody were isolated and stimulated *in vitro* with SGS (equivalent to 1.5 pairs of salivary glands) for 72 hours. For the control group, PBMCs were isolated from individuals living in a non-endemic area of Salvador, Bahia, Brazil and stimulated with SGS for 72 hours. The expression of (A) Ifit, (B) Irgm, (C) Stat1, and (D) CXCL9 was determined by real-time quantitative PCR. *** p<0.0001.

Despite a reduced number of IRG homologues in humans, some of the IFN-inducible genes modulated in the mouse upon SGS pre-immunization have homologues in humans [26]–[30]. In order to determine if the same genes were upregulated in humans naturally exposed to *Lu. intermedia* saliva, we isolated PBMCs from individuals living in Corte de Pedra, Brazil, an area endemic for *L. braziliensis* with active *Lu. intermedia* sand fly transmission [31]. Exposure to *Lu. intermedia* bites was determined based on a positive serology result for anti-*Lu. intermedia* SGS antibodies using a mean OD cutoff of 0.2711 (+/− 0.1006 SD) (Carvalho et al., unpublished data). Following PBMC isolation, cells were cultured in the presence or absence of *Lu. intermedia* SGS followed by mRNA isolation. PBMCs from these exposed individuals exhibited higher levels of Ifit1, Irgm, Stat1 and CXCL9 mRNA in response to SGS compared to PBMCs isolated from people living in a non-endemic area (Fig. 3). Similarly, supernatants from PBMCs of individuals living in an endemic area stimulated with SGS also produced significantly more CXCL9 protein as measured by ELISA compared to controls (data not shown). These data demonstrate that IFN-inducible genes are induced in response to SGS pre-sensitization in both the experimental model and in cells from human individuals pre-exposed to *Lu. intermedia* bites.

**Figure 4:**
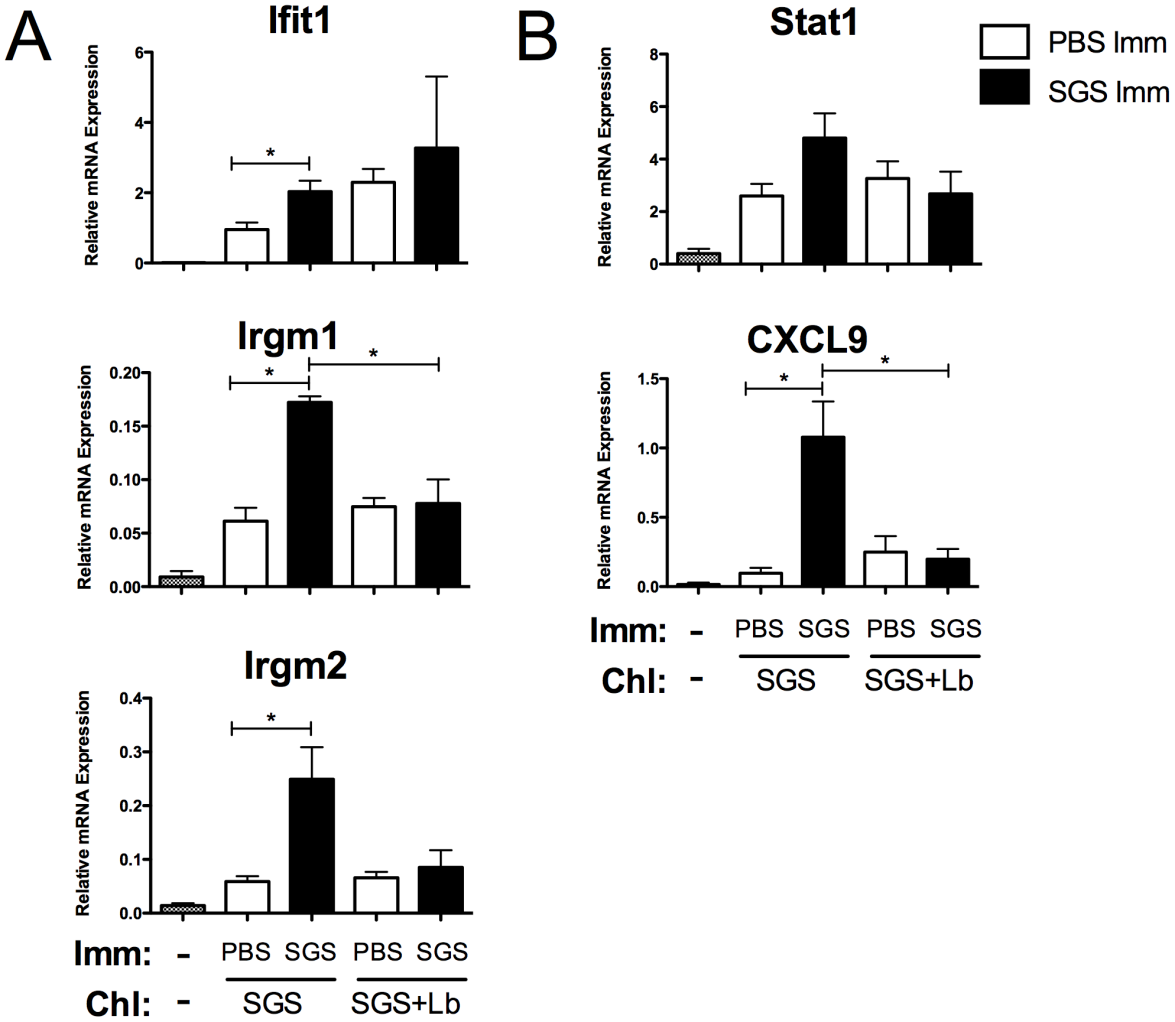
*L. braziliensis* infection prevents the upregulation of IFN-inducible genes due to SGS pre-immunization. BALB/c mice were inoculated 3 times in the right ear pinna every 2 wks with *Lu. intermedia* SGS and then challenged in the left ear 2 wks later with *Lu. intermedia* SGS plus 1×10^6^ *L. braziliensis* stationary phase promastigotes. The left ears were collected 2 wks after infection, homogenized and the expression of IFN-inducible genes such as (A) Ifit1, Irgm1 and Irgm2, and the expression of IFN-related genes like (B) Stat1 and CXCL9 was determined by real-time quantitative PCR. Relative mRNA expression normalized to the housekeeping gene HPRT is presented as the mean +SEM (n = 5 mice per group); * p<0.05 by Student's *t*-test.

Here, we show that SGS pre-immunization induces the expression of IFN-inducible genes, which are typically associated with a protective response as Irgm1^−/−^ animals are highly susceptible to *Leishmania* infection (mentioned as data not shown in [21]). However, pre-immunization with *Lu. intermedia* SGS has been reported to enhance *L. braziliensis* infection [12]. To evaluate the effect of the parasite on the local immune response induced by pre-immunization with SGS, mice were repeatedly pre-exposed to SGS or PBS and challenged with SGS in the presence or absence of *L. braziliensis* parasites. Gene expression profiling studies were carried out 2 weeks later. Mice that were pre-sensitized with SGS and challenged with SGS alone significantly upregulated the expression of Ifit1, Irgm1, Irgm2, Stat1 and CXCL9 compared to PBS pre-inoculated mice in line with our microarray data (Fig. 4). However, the mice that were pre-exposed to SGS and challenged with SGS and *L. braziliensis* did not significantly upregulate the expression of these genes compared to controls (Fig. 4 and Table S2). Taken together, these data suggest that *L. braziliensis* parasites modulate host gene expression at the site of infection creating a more hospitable environment for parasite establishment which is associated with increased lesion development.

## Discussion

Many studies have suggested the anti-saliva response against sand fly species such as *P. papatasi* or *L. longipalpis* is detrimental for the establishment of *Leishmania* infection. In contrast, the *Lu. intermedia* anti-saliva response does not prevent the development of disease, but rather may modulate the outcome of infection. Studies in a mouse experimental model have demonstrated that *L. braziliensis* infection alone induces a strong Th1 cell immune response with high levels of IFNγ and elevated numbers of IFNγ-producing CD4^+^ and CD8^+^ T cells in the dLN [32]–[34]. The strong protective immune response characterized by the presence of IFNγ was thought to correlate with the strong resistance to *L. braziliensis* infection [34]–[39]. Here, we show that repeated pre-immunizations with *Lu. intermedia* SGS alters the skin microenvironment and induces the expression of a variety of genes involved in the immune response, especially from the family of IFN-inducible genes. Genomic analysis of the skin of mice pre-immunized with SGS reveals an inflammatory setting with an increase in genes involved in immune responses including antigen presentation and cell signaling. Genes associated with Th1 cell immune responses such a CXCL9, a chemokine typically linked with the recruitment of Th1 cells, exhibited the greatest fold induction at >5 times over control mice. The IL-7R, also known to influence the Th1 cell immune response, was also elevated following SGS pre-exposure (this study and [40]–[42]). Of note, cytokines typically associated with a Th2 cell immune response such as Chi3l1 (Ym1), or regulatory cytokines such as IL-10Rα were also detected at higher levels in SGS pre-immunized mice.

In the periphery IFNγ binds to its receptor and initiates the JAK/STAT signaling pathway leading to the phosphorylation and translocation of STAT1 to the nucleus which induces the transcription of more than 2000 genes including effector molecules that suppress the growth and survival of intracellular pathogens (Phox, iNOS, IDO, NRAMP1, GTPases, Ifits and chemokines) [21]. Remarkably, several IFN-inducible genes as well as the IFN signaling molecule, STAT1, were upregulated at the site *Lu. intermedia* challenge in mice that were pre-exposed through immunization. For example, p47 GTPases such as Irgm1 (formerly Lrg47) and Irgm2 (Gtpi) and p65 GTPases such as GBP6 and GBP8 were expressed at high levels in pre-immunized mice.

Interestingly, IFN-inducible genes were similarly induced in SGS-stimulated PBMCs isolated from humans living in an area endemic for *L. braziliensis* with active *Lu. intermedia* sand fly transmission. It was not possible to perform skin biopsy in the human population studied due to ethical considerations; however, the expression of IFN-inducible genes in SGS-stimulated blood cells of individuals naturally exposed to sand fly bites was similar to that observed at the site of SGS challenge in mice. Collectively, these data demonstrate that the induction of IFN-inducible genes by SGS is also occurring in humans.

To our knowledge this is the first report demonstrating an induction in the expression of IFN-inducible GTPases in response to vector saliva. These products have been well characterized for their role in host defense against viruses but they also contribute to resistance against protozoans. Mice deficient for either Irgm1 or many of the other GTPases are highly susceptible to infection with *Toxoplasma gondii, Trypanosoma cruzi* and *Leishmania major*, and many mimic the dramatic susceptibility phenotypes seen in IFNγR-deficient mice [21]. It should be noted that IFN-inducible genes are turned on in response to IFNγ but type I IFNs may also contribute, although to a lesser degree [21]. In our analysis neither IFNγ or type I IFNs were elevated in cells from mice pre-sensitized with SGS but this may be a reflection of the time point analyzed (14 days post inoculation).

In this study mice were immunized with SGS to mimic one of the features of natural transmission of *Leishmania* where individuals are pre-exposed to several sand fly bites prior to deposition of parasites by the sand fly. A high dose of *L. braziliensis* promastigotes was co-inoculated in mice with SGS in an attempt to reproduce the cell recruitment rapidly observed at the site of infection after a sand fly bite. However, it is important to note that upon a blood meal, the sand fly is inoculating fewer parasites and also regurgitating many other factors including metacyclic promastigotes embedded in a proteophosphoglycan gel in a blood pool [2]. These factors are not all present during needle inoculation of the parasites and SGS. It is clear that further studies using natural sand fly infection will be required for a better understanding of the transmission dynamics during *Leishmania* infection.

Repeated exposures to *Lu. intermedia* SGS followed by challenge with *L. braziliensis* parasites in the presence of SGS leads to an enhanced disease compared to control mice (Fig. 1A and [12]). Interestingly, in this prior study the SGS pre-immunized mice challenged with *L. braziliensis* plus SGS, and analyzed two weeks later had a lower parasite load compared to mice not immunized with SGS, suggesting a transient protection conferred by SGS pre-immunization [12]. However, the trend was inversed from 3 weeks on and the SGS-immunized group showed increased lesion size and parasite load [12]. Additionally, the mice pre-immunized with SGS and subsequently infected with *L. braziliensis* plus SGS had lower levels of IFNγ to IL-4 ratios compared to mice inoculated with PBS and infected with *L. braziliensis* plus SGS at 2 weeks post infection. Thus, in that study, the highest levels of IFNγ were not associated with the decreased parasite numbers *in vivo* suggesting IFNγ is not the major factor contributing to parasite killing by macrophages at this time point. In our study, increased expression of IFN-inducible genes and of IFNγ was also not detected in the microarray performed in SGS pre-immunized mice 2 weeks post co-inoculation of *L. braziliensis* and SGS compared to PBS controls challenged with *L. braziliensis* plus SGS. In this and the previous study, the levels of IFNγ upon challenge with *L. braziliensis* and SGS were not elevated following SGS pre-immunization. Thus other factors may be involved in the transient control of parasite load observed by de Moura and colleagues [12]. Furthermore, higher concentrations of IFNγ are required for optimal parasite killing of *L. braziliensis* compared to *L. major* suggesting differences in the susceptibilities to IFNγ-mediated killing between different parasite strains [15]. Nevertheless, following *L. braziliensis* and SGS co-inoculation, both studies showed increased inflammatory lesions in the group pre-immunized with SGS compared to that injected with PBS.

This increase in disease severity to *L. braziliensis* infection in SGS pre-immunized mice, corresponds to a silencing of many of the genes turned on by SGS pre-sensitization, including IFN-inducible genes. This suggests that the parasite is actively modulating the host's immune response to the SGS. Interestingly, this observation is consistent with previous findings showing a decreased ratio of IFNγ/IL-4 production in the dLNs of mice pre-exposed to SGS and challenged with parasites [12]. In the same line, the same group further reported that challenge with *L. braziliensis* plus SGS after SGS pre-immunization also silenced CXCL10, another IFN-inducible gene [13]. Despite differences in the methodology used between these studies (air pouch model in the former studies and needle inoculation in the ear pinna in the current study), the outcomes are going in the same direction. In addition, the impact of SGS on the skin microbiome which was shown to influence skin immunity may also contribute to the phenotype observed [43]. We show here that there is an obvious benefit for the parasite to down-modulate the IRG system expressed in response to SGS pre-exposure to allow for parasite establishment. However, modulation of IFN-inducible genes is most likely not the only mechanism for enhancing disease. It is unclear how the parasite is altering the host's response to the SGS in the present study and this will require further investigation.

In conclusion, we have shown that in both humans and mice, an array of IFN-inducible genes were up-regulated in response to *Lu. intermedia* SGS pre-exposure. Interestingly, these genes were silenced when the parasite was present during the challenge. Given the marked changes in the skin microenvironment resulting from repeated exposures to *Lu. intermedia* SGS, and the different outcomes to *Leishmania* infection, understanding the relationship between pathogens and their homologous vectors is essential. Since SGS proteins from different sand fly species can either exacerbate or protect from disease, subsequent studies will aim to understand how the parasite is modulating SGS impact on the microenvironment [44]. This will help determine risk factors for disease development, markers of exposure and defining potential vaccine candidates.

## Supporting Information

Figure S1 **SGS pre-immunization does not modify cellular recruitment in response to** ***L. braziliensis*** **inoculation.** BALB/c mice were inoculated 3 times in the right ear every 2 wks with 1 pair of *Lu. intermedia* salivary glands and then challenged 2 wks later in the left ear with *L. intermedia* SGS. Ears were digested 2 wks post inoculation and cellular content was analyzed by FACS. Cell numbers are shown as the mean +SEM with 5 mice per group. Data are results from one experiment and representative of 2 individual experiments.
(TIF)

Table S1 **List of the primers used in this study to analyze gene expression by RT-PCR in mouse and human samples.**
(DOC)

Table S2 **List of the genes that were positively (>1.5 fold) or negatively (<1.5 fold) regulated at the site of parasite inoculation in mice immunized with SGS and subsequently infected with** ***L. braziliensis*** **plus SGS, compared to mice pretreated with PBS and infected with** ***L. braziliensis*** **plus SGS.** Contralateral ears were isolated for microarray analysis 2 weeks after parasite challenge. The data are presented as the fold change of mice pre-immunized with SGS (3 times every 2 weeks) over mice inoculated with PBS and challenged with *L. braziliensis* plus SGS. p-values<0.05: statistically significant. The values for IFN-inducible genes (below the IFN-inducible shaded line) are given but they did not vary >1.5 times and were not statistically significant between *L. braziliensis* samples that were pretreated with SGS or PBS.
(DOC)
