# Supplementary figures and images for: Repeated Exposure to Lutzomyia intermedia Sand Fly Saliva Induces Local Expression of Interferon-Inducible Genes Both at the Site of Injection in Mice and in Human Blood
Source: PLoS Negl Trop Dis. 2014 Jan 9;8(1):e2627. doi: 10.1371/journal.pntd.0002627 (PMC3888461; doi:10.1371/journal.pntd.0002627)

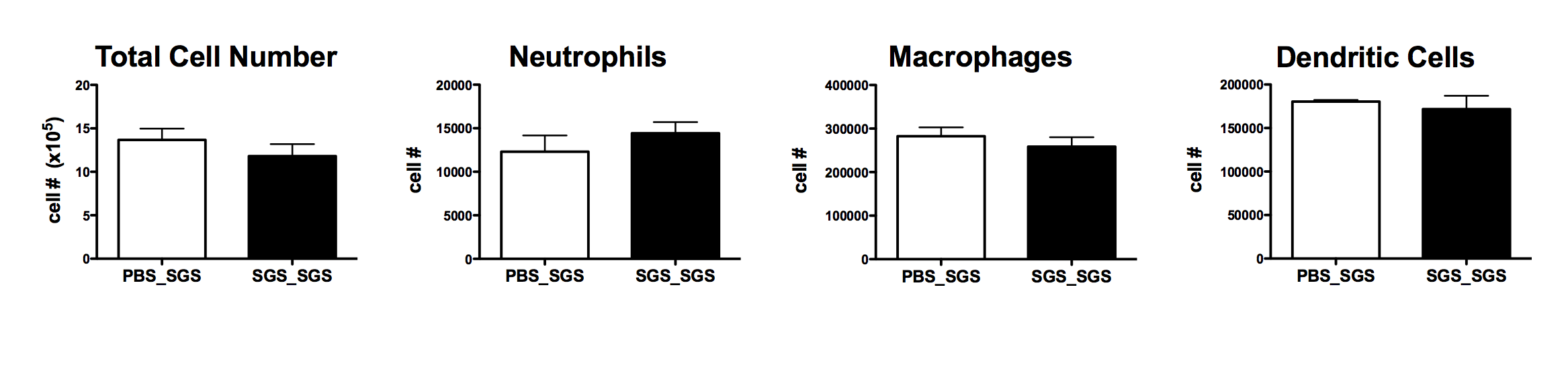

Supplement: Figure S1 — SGS pre-immunization does not modify cellular recruitment in response to L. braziliensis inoculation. BALB/c mice were inoculated 3 times in the right ear every 2 wks with 1 pair of Lu. intermedia salivary glands and then challenged 2 wks later in the left ear with L. intermedia SGS. Ears were digested 2 wks post inoculation and cellular content was analyzed by FACS. Cell numbers are shown as the mean +SEM with 5 mice per group. Data are results from one experiment and representative of 2 individual experiments. (TIF) [file pntd.0002627.s001.tif]
