# Supplementary material for: Repeated Exposure to Lutzomyia intermedia Sand Fly Saliva Induces Local Expression of Interferon-Inducible Genes Both at the Site of Injection in Mice and in Human Blood
Source: PLoS Negl Trop Dis. 2014 Jan 9;8(1):e2627. doi: 10.1371/journal.pntd.0002627 (PMC3888461; doi:10.1371/journal.pntd.0002627)
Supplement: Table S1 — List of the primers used in this study to analyze gene expression by RT-PCR in mouse and human samples. (DOC) [file pntd.0002627.s002.doc]

**TABLE S1. Primers**

| Gene | Sequences (5’ to 3’) | Species | Reference |
| --- | --- | --- | --- |
| *Cxcl9* (MIG) | F: TCCTTTTGGGCATCATCTTCC | Mo |  |
|  | R: TTTGTAGTGGATCGTGCCTCG |  |  |
| *Hprt* | F: GTT GGA TAT GCC CTT GAC | Mo |  |
|  | R: AGG ACT AGA ACA CCT GCT |  |  |
| *Ifit1* | F: CTGAGATGTCACTTCACATGGAA | Mo |  |
|  | R: GTGCATCCCCAATGGGTTCT |  |  |
| *Irgm1* | F: AAGGCCACTAACATCGAATCA | Mo |  |
|  | R: TGCCTTATCTCACTTAATACTCCTCA |  |  |
| *Irgm2* | F: CGGAGAGCTGTGGAGAGAGA | Mo |  |
|  | R: GCCATGTTTATGAAAAGTGTAAAAGT |  |  |
| *Stat1* | F: TCACAGTGGTTCGAGCTTCAG | Mo |  |
|  | R: GCAAACGAGACATCATAGGCA |  |  |
|  |  |  |  |
| *CXCL9* (MIG) | F: CCAAGGGACTATCCACCTACAATC | Hum |  |
|  | R: GGTTTAGACATGTTTGAACTCCATTC |  |  |
| *HPRT* | F: CCTGGCGTCGTGATTAGTGAT | Hum |  |
|  | R: AGACGTTCAGTCCTGTCCATAA |  |  |
| *IFIT1* | F: GCCTCCTTGGGTTCGTCTATA | Hum |  |
|  | R: TCAAAGTCAGCAGCCAGTCTCA |  |  |
| *IRGM* | F: GGAACTTGCCAGAGGTGATCTC | Hum |  |
|  | R: GCCTTACCCTCATGTCCTGTGT |  |  |
| *STAT1* | F: ATGTCTCAGTGGTACGAACTTCA | Hum |  |
|  | R: TGTGCCAGGTACTGTCTGATT |  |  |
|  |  |  |  |

1. Ma XZ, Bartczak A, Zhang J, et al. (2010) Proteasome inhibition in vivo promotes survival in a lethal murine model of severe acute respiratory syndrome. J Virology 84:12419-12428.

2. Charmoy M, Megnekou R, Allenbach C, et al. (2007) Leishmania major induces distinct neutrophil phenotypes in mice that are resistant or susceptible to infection. J Leuko Biol 82:288-299.

3. Hao S, Baltimore D (2009) The stability of mRNA influences the temporal order of the induction of genes encoding inflammatory molecules. Nat Immunol 10:281-288.

4. Simova J, Pollakova V, Indrova M, et al. (2011) Immunotherapy augments the effect of 5-azacytidine on HPV16-associated tumours with different MHC class I-expression status. British J Cancer 105:1533-1541.

5. Nishioka Y, Manabe K, Kishi J, et al. (2007) CXCL9 and 11 in patients with pulmonary sarcoidosis: a role of alveolar macrophages. Clin and Exp Immunol 149:317-326.

6. Abbasi F, Amiri P, Sayahpour FA, et al. (2012) TGF-beta and IL-23 gene expression in unstimulated PBMCs of patients with diabetes. Endocrine 41:430-434.

7. Ye S, Pang H, Gu YY, et al. (2003) Protein interaction for an interferon-inducible systemic lupus associated gene, IFIT1. Rheumatol (Oxford) 42:1155-1163.

8. Xu H, Wu ZY, Fang F, et al. (2010) Genetic deficiency of Irgm1 (LRG-47) suppresses induction of experimental autoimmune encephalomyelitis by promoting apoptosis of activated CD4+ T cells. FASEB J : 24:1583-1592.

9. Zhu B, Kuriakose JA, Luo T, et al. (2011) Ehrlichia chaffeensis TRP120 binds a G+C-rich motif in host cell DNA and exhibits eukaryotic transcriptional activator function. Infect and Immun 79:4370-4381.
