# Supplementary material for: Repeated Exposure to Lutzomyia intermedia Sand Fly Saliva Induces Local Expression of Interferon-Inducible Genes Both at the Site of Injection in Mice and in Human Blood
Source: PLoS Negl Trop Dis. 2014 Jan 9;8(1):e2627. doi: 10.1371/journal.pntd.0002627 (PMC3888461; doi:10.1371/journal.pntd.0002627)
Supplement: Table S2 — List of the genes that were positively (>1.5 fold) or negatively (<1.5 fold) regulated at the site of parasite inoculation in mice immunized with SGS and subsequently infected with L. braziliensis plus SGS, compared to mice pretreated with PBS and infected with L. braziliensis plus SGS. Contralateral ears were isolated for microarray analysis 2 weeks after parasite challenge. The data are presented as the fold change of mice pre-immunized with SGS (3 times every 2 weeks) over mice inoculated with PBS and challenged with L. braziliensis plus SGS. p-values<0.05: statistically significant. The values for IFN-inducible genes (below the IFN-inducible shaded line) are given but they did not vary >1.5 times and were not statistically significant between L. braziliensis samples that were pretreated with SGS or PBS. (DOC) [file pntd.0002627.s003.doc]

**TABLE S2. Genes with differential regulation to SGS pre-exposure upon *L. braziliensis* infection**

| **Gene Symbol** | **Description** | **Fold Change** | **p-value** |
| --- | --- | --- | --- |
| Pyy | Peptide YY | 1.71 | 0.011 |
| Sprr1b | Small proline-rich protein 1B | -1.99 | 0.032 |
| Stfa3 | Stefin A3 | 1.61 | 0.023 |
| Top2a | Topoisomerase (DNA) II alpha | 1.62 | 0.024 |
| **IFN-inducible genes** | | | |
| Gbp6 | Guanylate-binding protein 6 | 1.18 | 0.47 |
| Gpb8 | Guanylate-binding protein 8 | 1.10 | 0.71 |
| Ifit1 | IFN-induced protein | 1.17 | 0.26 |
| Iigp1 | IFN-inducible GTPase | 1.51 | 0.45 |
| Irgm1 | Immunity-related GTPase | 1.24 | 0.30 |
| Irgm2 | Immunity-related GTPase | 1.24 | 0.48 |
